# Supplementary material for: Comprehensive Annotation and Functional Exploration of MicroRNAs in Lettuce
Source: Front Plant Sci. 2021 Dec 24;12:781836. doi: 10.3389/fpls.2021.781836 (PMC8739914; doi:10.3389/fpls.2021.781836)
Supplement: Supplementary file 2 [file Data_Sheet_8.ZIP › GSM2230750.plot/Lsa-miRN1732_Lsat_1_v5_gn_6_32121.1_3193_TPlot.pdf]

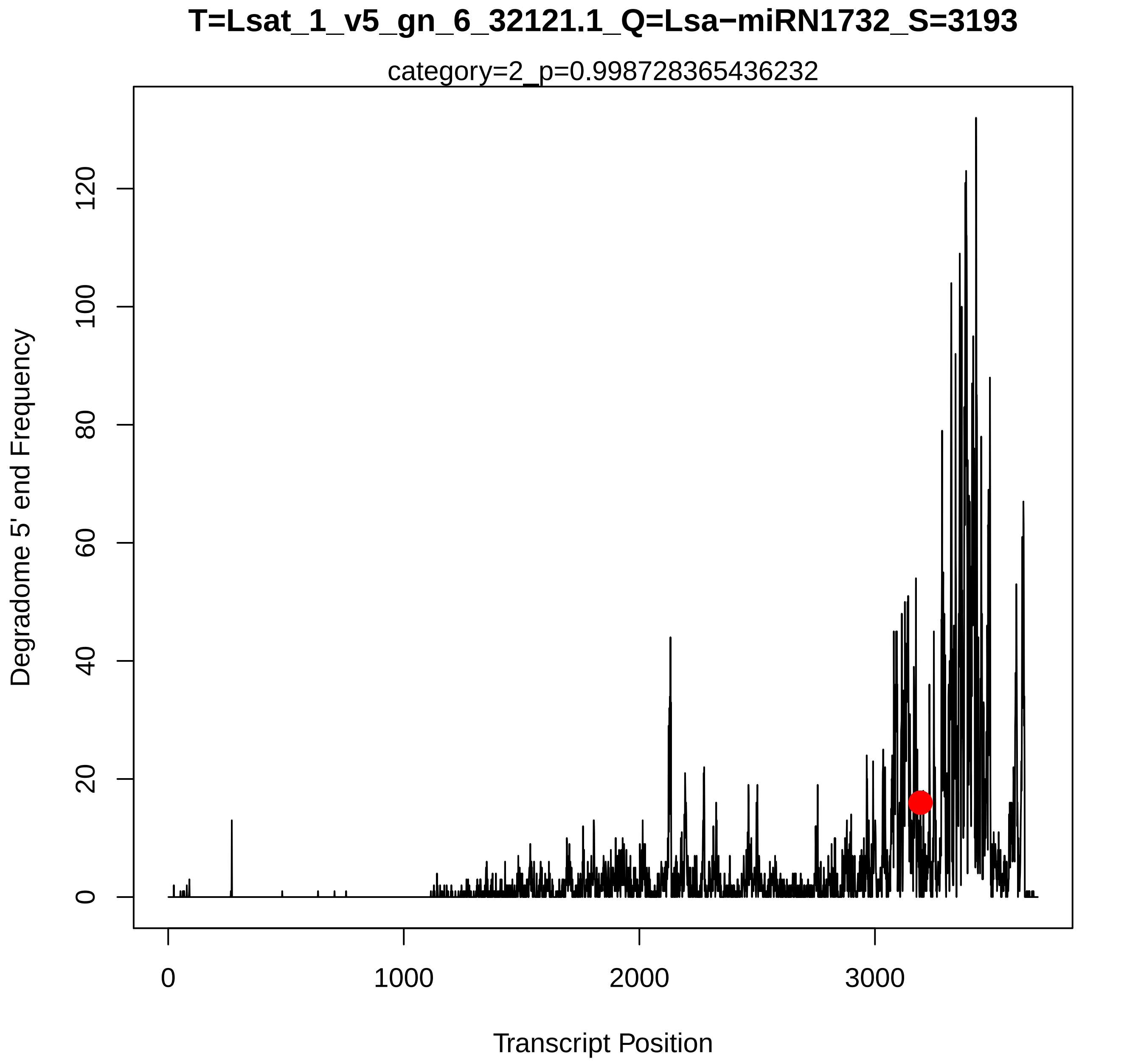

T=Lsat_1_v5_gn_6_32121.1_Q=Lsa−miRN1732_S=3193
category=2_p=0.998728365436232
Degradome 5' end Frequency
0
20
40
60
80
100
120
0
1000
2000
3000
Transcript Position
